# Exploring Overlapping Functional Units with Various Structure in Protein Interaction Networks

**DOI:** 10.1371/journal.pone.0043092

**Published:** 2012-08-20

**Authors:** Xiao-Fei Zhang, Dao-Qing Dai, Le Ou-Yang, Meng-Yun Wu

**Affiliations:** Center for Computer Vision and Department of Mathematics, Sun Yat-Sen University, Guangzhou, China; Technical University of Madrid, Italy

## Abstract

Revealing functional units in protein-protein interaction (PPI) networks are important for understanding cellular functional organization. Current algorithms for identifying functional units mainly focus on cohesive protein complexes which have more internal interactions than external interactions. Most of these approaches do not handle overlaps among complexes since they usually allow a protein to belong to only one complex. Moreover, recent studies have shown that other non-cohesive structural functional units beyond complexes also exist in PPI networks. Thus previous algorithms that just focus on non-overlapping cohesive complexes are not able to present the biological reality fully. Here, we develop a new regularized sparse random graph model (RSRGM) to explore overlapping and various structural functional units in PPI networks. RSRGM is principally dominated by two model parameters. One is used to define the functional units as groups of proteins that have similar patterns of connections to others, which allows RSRGM to detect non-cohesive structural functional units. The other one is used to represent the degree of proteins belonging to the units, which supports a protein belonging to more than one revealed unit. We also propose a regularizer to control the smoothness between the estimators of these two parameters. Experimental results on four S. cerevisiae PPI networks show that the performance of RSRGM on detecting cohesive complexes and overlapping complexes is superior to that of previous competing algorithms. Moreover, RSRGM has the ability to discover biological significant functional units besides complexes.

## Introduction

A key task of postgenomic systems biology is to cluster proteins and their interactions into functional units (groups) within a living cell, which further facilitates unveiling the complex machinery of cells functional organization [1]. The recent developments of high-throughput experimental techniques for delineating protein-protein interactions and modern data warehousing techniques for collecting available data make it possible to reveal functional units in protein-protein interaction (PPI) networks in genomic scale.

**Figure 1:**
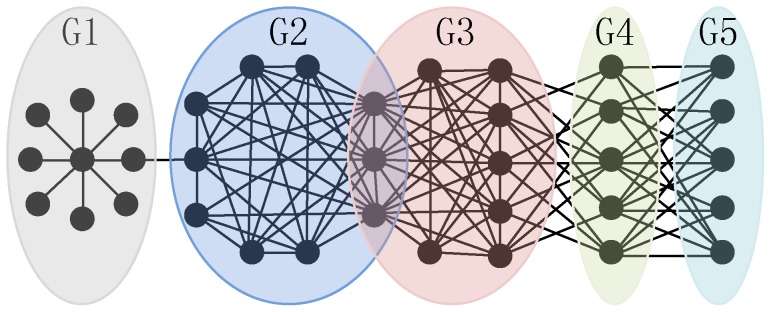
An example PPI network that includes overlapping and different structural functional groups. Group G1 is a spoke model in which all spoke proteins interact with the hub protein. Groups G2 and G3 are two cohesive groups that have more interactions within themselves and fewer with the rest of the network. And there are three proteins shared by these two groups. Group G4 is a non-cohesive group of which the members similarly interact with proteins in groups G3 and G5, but not among themselves. Group G5 is another non-cohesive group in which the proteins only have interaction with proteins in group G4, but not among each other.

The typical approaches to detect functional units in PPI networks resort to identifying cohesive protein complexes or functional modules [2]–[4] (Here, we do not distinguish protein complexes from functional modules because the underlying PPI networks that we are using for functional units detection do not provide temporal and spatial information which is important for differentiating these two concepts). Based on the widely accepted definition of protein complex that a complex in the PPI network is a cohesively connected subnetwork which has more interactions within itself and fewer with the rest of the network (e.g., groups G2 and G3 in Figure 1), various computational algorithms have been successfully proposed to mine these cohesive complexes [5]–[8]. The proteins generally perform different biological functions by interacting with distinct partners [9], [10], thus real protein complexes in the PPI network often overlap such that the proteins simultaneously belong to several functional groups (e.g., the common three proteins shared by groups G2 and G3 in Figure 1). Clearly, the traditional protein complexes revealing algorithms that do not support overlapping complexes are not able to present the biological reality. Hence, much recent attention has been focused on detecting overlapping protein complexes [11]–[17].

The definition of protein complex corresponds to the concept of modular organization of PPI networks [18], which is a typical principle of biological networks [1], [19]. However, Wang and Zhang [20] recently suggest that cohesive modules may originate as an evolutionary byproduct without much biological significance, thus may be not able to completely depict various functional units in PPI networks. Motivated by this result, two algorithms have been developed to mine other structural functional units besides cohesive functional modules [21], [22]. Experimental results of their studies show that besides salient cohesive functional modules, other functional significant non-cohesive structures (e.g., groups G4 and G5 in Figure 1) also exist in the PPI networks. The modularity-based method of Pinkert *et al.* [21] has limited application in revealing functional units not only for the reason of resolution limit [23] but also over-split phenomena [22]. Other biological meaningful units (e.g., spoke model [24] in Figure 1) may be neglected by Jiao *et al.*s algorithm which is just designed to mine bi-sparse and cohesive modules based on edge density [22]. Thus, it needs to develop an effective algorithm that is able to explore overlapping and various structural functional units in PPI networks.

Recently, the random graph models have been proposed to model PPI networks in terms of a random process to generate the networks [25], [26]. Besides just generating synthetic data, several recent studies have explored the new applications of random graph models in biological network analysis [27]–[30]. The recent advances in application of random graph models in biological networks motivate us to explore their further application in mining functional units in PPI networks.

Outside of biology, Newman and Leicht [31] describe a mixture model to detect structure in networks by defining a structure as a group of nodes that all have similar patterns of connections to others. Ball *et al.* [32] recently introduce a random graph model to detect overlapping cohesive communities in social networks based on the concept of link communities [19]. Motivated by these two prominent works in physics, we present a new regularized sparse random graph model (RSRGM) to explore functional units in PPI networks. By applying our model to four S. cerevisiae PPI networks, we show that RSRGM not only gives competitive results with the state-of-the-art algorithms on detecting cohesive complexes and overlapping complexes, but also is able to discover other non-cohesive structural functional units.

## Methods

In this section, we outline the main idea of our algorithm. First we develop a sparse random graph model to describe the generation process of the PPI network, which is mainly determined by two model parameters: one for presenting the propensities of proteins belonging to groups, the other for defining the structure of functional units. We also introduce a regularizer to control the smoothness between estimators of these two parameters. Finally, we develop a new smooth regularized sparse random graph model and use it to explore functional units in PPI networks by estimating the two parameters.

### A sparse random graph model for PPI networks

Given a PPI network, we attempt to define its generation process using a random graph model. We first model a PPI network with 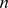 proteins as a direct graph with adjacent matrix 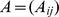, where 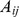 indicates whether there is a (direct) link from protein 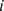 to protein 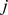. Instead of the undirected graph presented in the general approaches, we use the directed graph since it is somewhat simpler in our model. We specify that proteins 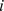 and 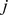 are connected if and only if there are a link from protein 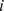 to protein 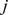 and a link from protein 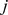 to protein 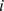 simultaneously. Hence we set 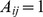 and 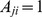 if proteins 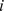 and 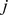 are connected, and 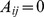 and 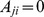 otherwise. Even though we model the PPI network as directed graph, the adjacent matrix 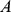 is symmetric in essence.

Suppose the proteins of the PPI network fall into 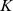 functional units. Similar to [32], we introduce 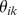 as the propensity of protein 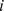 belonging to group 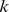, a higher value of 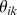 indicates that protein 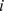 is more likely in group 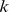 and a lower value of 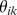 means that protein 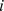 is less likely in group 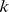. Note that for a protein 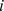, it may obtain high value of 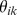 on more than one group, thus our model essentially supports overlaps among functional units. Similar to [31], we also introduce 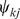 to represent the propensity that there is a (directed) link from a particular protein in group 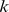 to protein 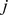. In effect 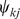 represents the preferences of proteins in group 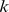 about which other proteins they link to. By these preferences, in this study a functional unit is defined as a group of proteins that all have similar patterns of connections to others. Note that, unlike the definitions of cohesive protein complexes [2] and bi-sparse modules [22], besides this rather broad and flexible definition, we do not make strict assumption of the structure of functional units we explore. Thus, the functional units we reveal cover a wide variety of structures including the cohesive complexes as a special case of which the members similarly interact among themselves.

Let 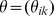 be the protein-group membership matrix and 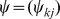 be the group-protein preference matrix. By the definitions of 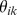 and 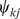, 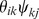 is assumed to be the likelihood that there is a link from protein 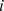 to protein 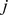 in terms of group 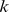 and 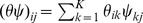 is the total likelihood in terms of all the 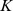 groups. Similar to [30] and [32], we assume the random number of links from protein 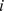 to protein 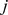 is independently from Poisson distribution with mean 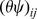. Note that this means that the network generated by our model is a technically multi-graph with self-links, which is unrealistic for PPI networks. But it greatly simplifies the mathematical developments and is also allowed by Ranola *et al.* [30] and Ball *et al.* [32]

In practice, it is well known that a protein in any particular PPI network usually belongs only to one or several functional units, seldom belongs to all considered units, and proteins in a considered unit usually connect only to a part of proteins in the whole PPI network but seldom connect to all proteins. Thus, we place independent exponential distribution priors over each element of 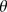 and 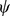 with rate parameter 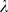, which indirectly imposes sparse restriction on the protein-group membership matrix and the group-protein preference matrix. The sparse restriction will lead all elements in some columns of 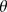 and rows of 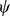 to be 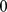 simultaneously, and hence the corresponding irrelevant groups disappear automatically. Therefore, it not only has good biological interpretation but also presents a method to determine the value of parameter 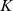, the number of functional units in the considered PPI network.

The quantities in our random graph model can be classified into three classes: observed adjacent matrix 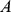 of the PPI network, model parameters 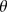 and 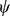, and hyperparameter 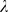. Given the rate parameter 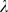, our model generates a PPI network with a given number 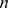 of proteins and a given number 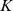 of functional units as follows:

• For each protein 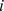 and group 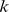, draw protein-group membership 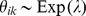 with the probability:
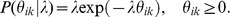
(1)
• For each group 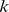 and protein 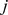, draw group-protein preference 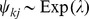 with the probability:
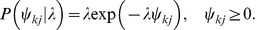
(2)
• For each pair of proteins 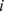 and 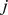, sample the value of their interaction 
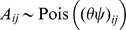
 with the probability:
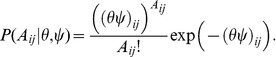
(3)

Under the introduced random graph model, the joint probability of a graph with adjacent matrix 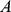 and the model parameters 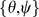 can be written as follows:
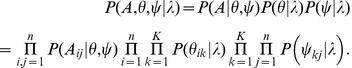
(4)


For an observed PPI network, we estimate the values of 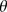 and 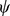 by maximum the joint probability of Equation (4). By taking Equations (3), (1) and (2) into Equation (4), taking the negative logarithm and dropping constants, we obtain the objective function of the sparse random graph model (SRGM):
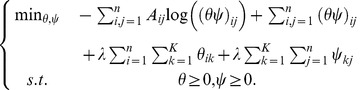
(5)


Here 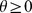 and 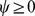 means each element 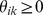 and 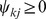.

### A smooth regularizer

If there is a link from protein 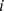 to protein 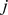, that is 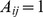, we can use 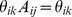 as an estimator of 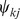 without considering other information besides 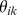 and 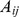. This is because we have defined 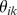 as the propensity of protein 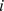 belonging to group 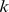 and 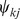 as the propensity of having a link from a protein in group 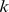 to protein 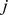. Hence, if protein 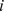 obtains a high value of 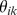 and 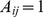, protein 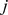 will obtain a high value of 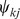, and vice versa. Similarly, we can use 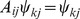 as an estimator of 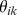 without considering other information besides 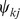 and 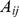. Thus, ideally, for two connected proteins 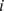 and 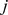, they tend to obtain similar values of 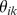 and 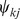.

We introduce the following regularizer to quantify the smoothness between the estimators of 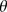 and 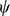 on the PPI network:
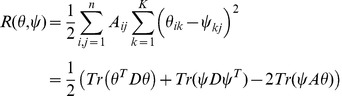
(6) where 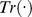 denotes the trace of a matrix, and 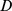 is a diagonal matrix with 
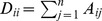
. We aim to minimize 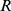 with respect to 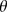 and 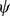 so that if proteins 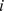 and 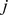 are connected, 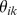 and 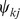 will be close to each other for 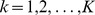.

### Regularized sparse random graph model

#### Objective function of regularized sparse random graph model

By combining the introduced smooth regularizer (6) with the objective function of sparse random graph model (5), we present a new model, named as regularized sparse random graph model (RSRGM), as follows:
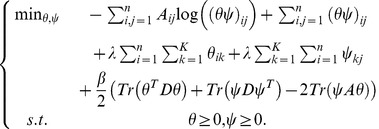
(7)


Here 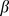 is a balance parameter between objective function of SRGM (5) and smooth regularizer (6).

#### Solution to regularized sparse random graph model

To optimize 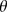 and 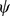, we follow the multiplicative updating rule [33] which is widely used to solve non-negative constrained optimization problem and involves consecutive updates of 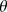 and 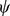 until a stopping criterion has been satisfied.

By the multiplicative updating rule, we obtain the following two updating formulas for 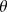 and 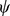, respectively:
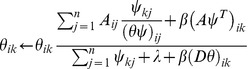
(8) and 
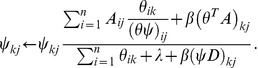
(9)


The updating formulas (8) and (9) are able to guarantee the nonnegativity of estimators of parameters. That is, if we initialize 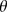 and 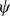 with nonnegative values, the elements of 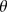 and 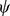 are always nonnegative during iteration. For the detailed inference of the two updating rules, please refer to Text S1.

#### From protein-group membership matrix to functional units

Different from previous algorithms detecting protein complexes by hard clustering, each element 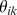 of 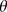 denotes the degree of protein 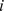 belonging to group 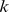. Thus we obtain functional groups from real-value of 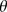 by assigning a protein to a group if its membership propensity for that group exceeds a given threshold 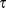:
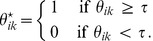
(10)


Here 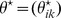 is the protein-group indication matrix of which 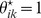 represents protein 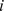 is in discovered group 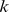 and 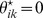 represents protein 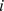 is not in discovered group 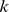. Since 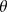 is sparse, all elements of many columns of 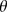 are 0, and hence the groups corresponding to these columns disappear. Here, similar to [16], we also filter out the detected groups of which the number of members is less than three, that is we delete the columns of 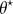 that contain at most two elements of 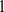.

#### Final algorithm

**Figure 2:**
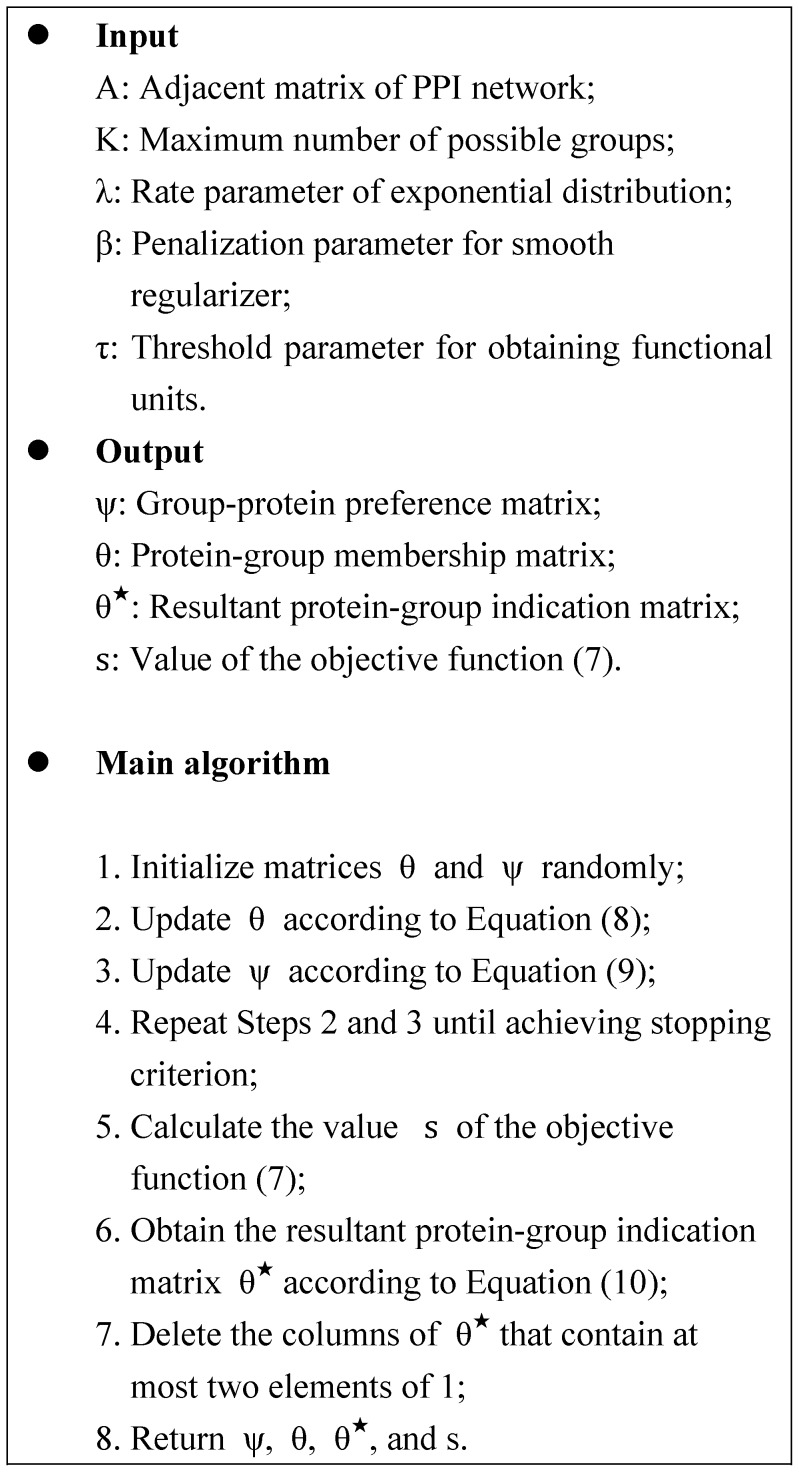
Summary of RSRGM for discovering protein functional units.

We summarize the detailed algorithm for discovering various structural functional units in the PPI networks via regularized sparse random graph model (RSRGM) in Figure 2. In this study, we limit a maximum of 150 iterations when updating 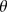 and 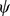 using Equations (8) and (9) for practical application purpose, although it frequently converges before this point is reached. Since the objective function of (7) is nonconvex, which will lead to a local minimum by the multiplicative updating rule. To guard against the possibility of getting stuck in a local minimum, we repeat the entire calculation 50 times with random initial conditions and choose the result that gives the lowest value of objective function of (7).

## Results

In this section, we first compare RSRGM to the previous competitive algorithms for detecting cohesive protein complexes and overlapping complexes. We then test the effect of RSRGM on unveiling other non-cohesive structural functional units. As we know, no common precise definition of cohesive complexes has been agreed upon. For simplicity, we differentiate the cohesive protein complexes and non-cohesive functional units based on density (where the density of a functional unit with 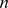 proteins is defined as the total number of its internal interactions, divided by 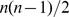). The identified functional units with density above 0.1 are viewed as cohesive complexes, and functional units with density below 0.1 are viewed as non-cohesive functional units in this study.

### Datasets

#### PPI networks

We concentrate our analysis on two experimental yeast PPI networks [34], [35], a combined computational interaction map [36] and the entire set of physical interactions in yeast from BioGRID [37]. Here we refer to these as Gavin, Krogan, Collins and BioGRID networks. The Gavin, Krogan and Collins networks are extracted from BioGRID database with Pubmed ID 16429126, 16554755 and 17200106, respectively. The BioGRID network is downloaded from version 3.1.88 and contains all physical interactions that involve yeast proteins only. Self-interactions, redundant interactions and interactions involving proteins of which systematic names are not available are filtered out from all the four networks. In this study, for simplicity, we just analyze the largest connected component of each network. Table 1 presents the number of proteins, the number of interactions, the average clustering coefficient, the average number of neighbors and the density of each PPI network.

**Table 1:** Topological characteristics of used PPI networks.

Network	# proteins	# interactions	cc	avNeighbors	density
Gavin	1359	6451	0.4196	9.49	0.0070
Krogan	2559	7031	0.1947	5.50	0.0021
Collins	1004	8319	0.6478	16.57	0.0165
BioGRID	5850	68312	0.2622	23.35	0.0040

Here cc denotes the average clustering coefficient of network, avNeighbors denotes the average number of neighbors of each protein.

#### Gold standard protein complexes

In order to evaluate the performance of our model on detecting cohesive protein complexes, we derive two gold standard complexes from MIPS [38] and SGD [39] databases, respectively (Table S1). We utilize the 220 filtered yeast protein complexes from MIPS which is the same set used by Brohée and Van Helden [40]. Since complexes in MIPS database are incomplete, we also use an additional independent reference set for validation. This set is generated from complexes in the SGD database as the procedure described by Nepusz *et al.* [16]. The SGD annotations and the cellular component ontology used to generate the SGD reference complexes are downloaded from Gene Ontology database [41] on 24 April 2012. To avoid selection bias, for both two reference sets we only consider complexes containing at least 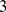 and at most 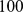 proteins with respect to each PPI network. Table 2 lists the number of complexes, the number of proteins covered by these complexes and the number of proteins shared by more than one complex for the four PPI networks considered in this study.

**Table 2:** Statistics of gold standard protein complexes.

Network	Reference database	# complexes	# proteins	# proteins in 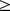 2 complexes
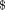 Total	MIPS	220	1095	300
	SGD	324	1340	293
Gavin	MIPS	94	537	142
	SGD	118	542	136
Krogan	MIPS	119	601	193
	SGD	168	790	197
Collins	MIPS	64	437	91
	SGD	81	426	116
BioGRID	MIPS	157	1010	279
	SGD	242	1217	262

Here 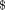 denotes the statistics of the total complexes of each reference database which are not mapped into PPI networks and filtered by size.

#### GO classification and annotations

We use Gene Ontology [41], a widely adopted gold standard system, as the data sources of functional classification and annotations to test functional homogeneity of non-cohesive functional units revealed by our approach. The Gene Ontology file including three ontologies (biological process, cellular component, and molecular function) and the GO annotations of S.cerevisiae are obtained from the Gene Ontology database on 24 April 2012.

### Evaluation measures

#### Metrics for evaluating protein complexes detection

We use three independent quantity measures to assess the similarity between a set of predicted protein complexes and a set of reference complexes (Text S2). The first one is accuracy (Acc) which is defined as the geometric average of sensitivity (Sn) and positive predictive value (PPV) [40]. Here note that we use the new definition of PPV introduced by Xie *et al.* [42] which is more suitable for evaluating overlapping clusters. The other two measures we use are the Jaccard and PR metrics introduced by Song and Singh [43].

#### Metrics for evaluating overlapping complexes detection

We use precision, recall and F-score to quantitatively evaluate the performance of different algorithms on detecting proteins shared by multiple protein complexes. Let 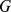 be the set of proteins shared by more than one complex in the gold standard database and 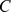 be set of proteins presented in more than one predicted complex. Then precision, recall and F-score are defined as follows:
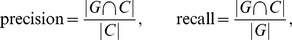


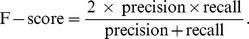



#### Metrics for evaluating non-cohesive functional units detection

We evaluate the unveiled non-cohesive functional units based on functional homogeneity in terms of GO annotations. The statistical significance of the occurrence of a non-cohesive functional unit with respect to a given functional annotation is computed by the following hypergeometric distribution:
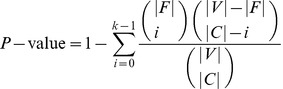
(11)where a revealed functional unit 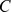 contains 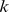 proteins annotated with GO function 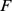 which associates with 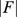 proteins, and the whole organism contains 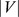 proteins. Here, we perform GO enrichment analysis by computing the P-value using GO Term Finder [44].

### Detecting cohesive protein complexes

#### Effect of parameters

There are four parameters 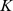, 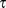, 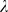 and 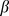 in our algorithm. 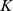 is the supposed maximum number of possible functional units in the PPI network, and we set 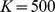 for Gavin, Krogan and Collins networks, and 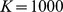 for BioGRID network. The parameter 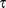 is the threshold used to obtain the resultant functional groups from protein-group membership matrix, and we experimentally find that a value of 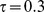 usually gives the reasonable results on the four networks. The parameters 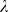 and 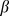 are two key parameters of our method, and we focus on studying the effect of these two parameters on detecting protein complexes. Particularly, we run RSRGM on the four yeast PPI networks with different combination values of 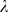 (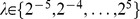) and 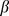 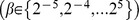, and evaluate the complexes identified by RSGNM using reference complexes in MIPS and SGD databases in terms of the three measures introduced above (Acc, Jaccard and PR).

**Figure 3:**
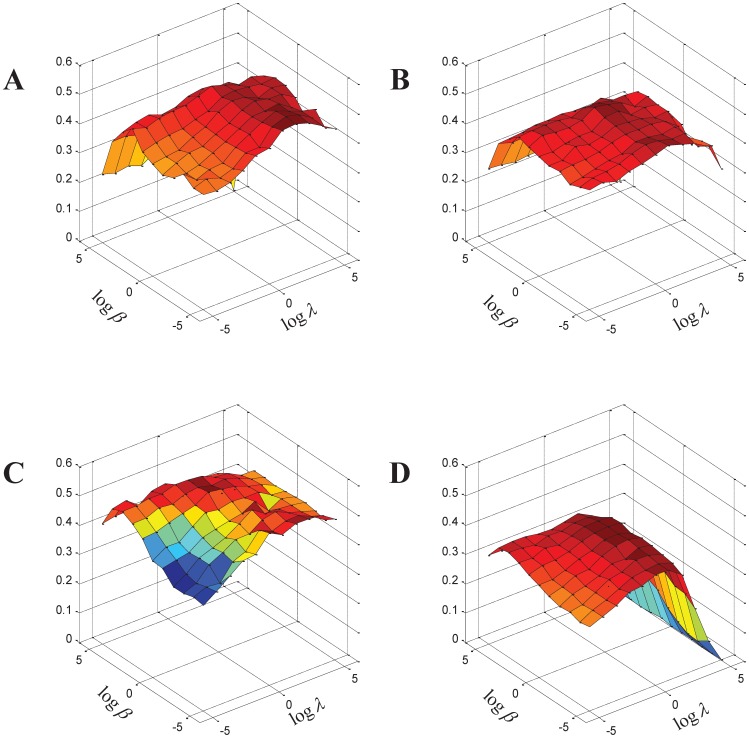
Performance of RSRGM on detecting complexes with respect to different values of 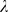
 **and** 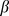 **.** The x-axis denotes the value of 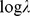, the y-axis denotes the value of 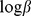, and the z-axis denotes the harmonic mean of the three measures scores of both MIPS and SGD reference complexes. (A) Gavin network. (B) Krogan network. (C) Collins network. (D) BioGRID network.

Figure 3 shows the harmonic mean of six scores (three different measure scores (Acc, Jaccard and PR) of the two different gold standard databases (MIPS and SGD)) with respect to various values of 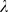 and 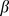 on the four PPI networks. In general, for a fixed value of 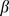, the harmonic mean scores increase initially and decreases after obtaining maximum with the increasing of the value of 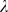. Similarly, for a fixed value of 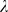, the harmonic mean scores increase initially and decreases after obtaining maximum when the value of 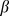 increases. This result shows the effectiveness of these two parameters since both of them contribute to improving the performance of RSRGM.

From this figure, we also find maximal value of harmonic mean of the six scores are obtained when 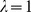 and 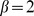 for Gavin network, 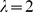 and 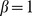 for Krogan network, 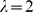 and 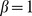 for Collins network, and 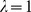 and 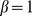 for BioGRID network. In the following study, unless otherwise stated, the resulting functional units (both cohesive complexes and non-cohesive functional units) identified by RSRGM are obtained with these optimal values of parameters for the four PPI networks.

#### Comparative evaluation on detecting complexes

To test the effectiveness of RSRGM on detecting protein complexes, we compare it to CFinder [11], ClusterOne [16], CMC [13], MCL [5], MCODE [6], MINE [14] and SPICi [8]. The simple description and detailed parameter settings of each algorithm are listed in Text S3. Note that for all considered methods, as RSGNM, we discard complex candidates with size less than three.

**Figure 4:**
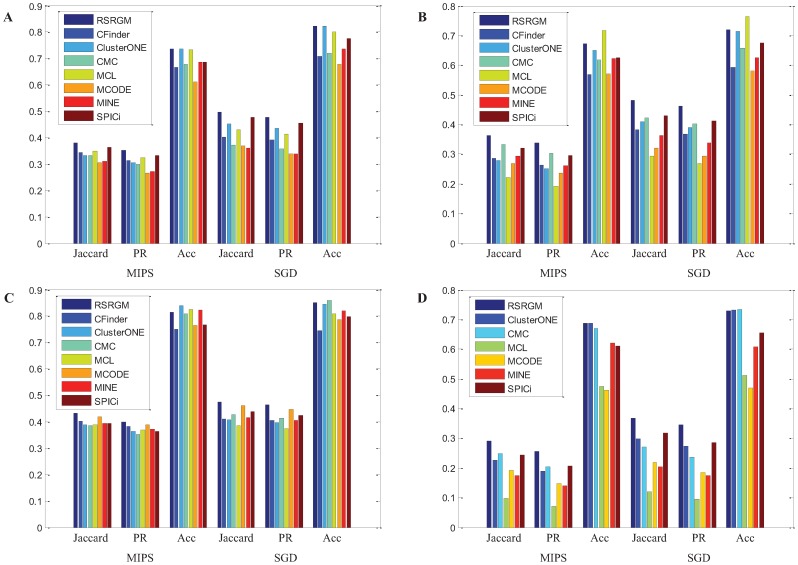
Benchmark results of various protein complexes detection algorithms on the four PPI networks. (A) Gavin network. (B) Krogan network. (C) Collins network. (D) BioGRID network.

Figure 4 graphically shows the comparative performance of considered algorithms with the three evaluation measures (Acc, Jaccard and PR) using both MIPS and SGD reference complexes on the four PPI networks. Note that since CFinder can not successfully analyze BioGRID network in 48 hours, we do not present corresponding results in this figure. We observe that the relative performance of these algorithms change depending on the networks under consideration, and none of them clearly dominates the other approaches in terms of all the three measures and both two gold standards. However, RSRGM performs better than other approaches on all the four networks in terms of Jaccard and PR measures. In most cases, RSRGM works as the best two methods with Acc metric. These results show that even though RSRGM is not just devised to detect cohesive protein complexes, it has competitive performance with the state-of-the-art algorithms at this task.

#### Comparative evaluation on detecting overlapping complexes

Various algorithms based on different principles have been developed to discover overlapping protein complexes. Thus besides comparing theses algorithms using the overlapping gold standard complexes as analyzed above, it is also interesting to directly compare RSRGM to these algorithms on revealing proteins shared by multiple complexes. Here, we focus on the competitive algorithms that are also able to handle overlaps: CFinder [11], ClusterONE [16], CMC [13] and MINE [14]. Note MCODE considered in this study is also able to produce overlapping complexes by executing the fluffing phase, but experimental results show that it performs more better on detecting complexes when fluffing is turned off. Thus we do not take it into account.

**Figure 5:**
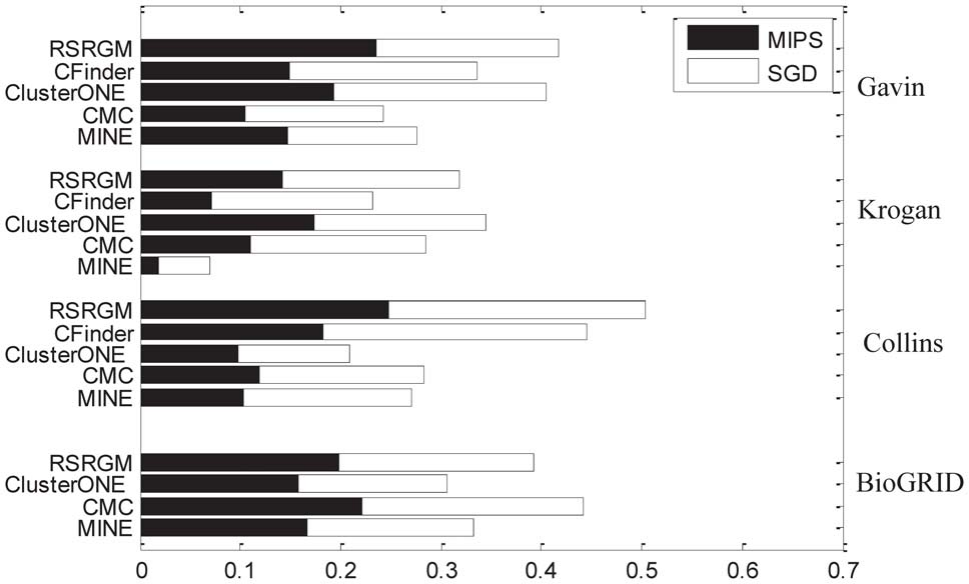
Comparative results of different algorithms on revealing proteins shared by multiple complexes. The total height of each bar is the composite value of F-scores on both MIPS and SGD databases for each algorithm.

In this experiment, we also use MIPS and SGD databases as gold standard reference complexes, and proteins shared by more than one complex in each database is the reference of multi-complex proteins. We present the F-scores in terms of the MIPS and SGD reference complexes in Figure 5; larger scores are better, and the sum of the two scores corresponding to the two difference databases is a composite score. Note that we do not present the corresponding results of CFinder on BioGRID network since it can not analyze this network in 48 hours. Experimental results on the four PPI networks show that the five approaches have complimentary strength in revealing multi-complex proteins when analyzing networks with different topological characteristics. ClusterONE outperforms the other four algorithms on Krogan network and CMC works as the best one on BioGRID network. We also observe that RSRGM performs better than the other four algorithms on Gavin and Collins networks and obtain the second highest composite scores on the other two networks. Experimental results show that compared to the previous competing algorithms, RSRGM is less sensitive to the change of topological characteristics of the network under consideration and is competitively accurate for revealing proteins shared by more than one complex. For detailed results of precision and recall, see Table S2.

### Detecting other non-cohesive structural functional units

Besides discovery of cohesive protein complexes, RSRGM is also developed to reveal other non-cohesive functional units of which the members seldom interact with each other but have the similar interactions with the rest of network. In this section, we study whether the non-cohesive functional units widely exist in PPI networks and represent biology. We evaluate the biological significance of the non-cohesive functional units identified by RSRGM in terms of the P-value of hypergeometric distribution calculated with GO Term Finder [44].

Table 3 presents the total number of identified functional units and the corresponding proteins, the number of non-cohesive units and the proteins they cover, and the number of GO significant non-cohesive units (of which the P-value is lower than 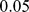 for at least one of the three ontologies: biological process, cellular component, and molecular function) and the proteins they involve. We can see that the proportion of non-cohesive functional units changes according to the topological characteristics of the network we consider. For Collins network which has high values of average cluster coefficient, average number of neighbors and network density, we find that among the total 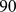 identified units, there are only 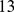 non-cohesive units. On the contrary, for Krogan network which has low values of average cluster coefficient, average number of neighbors and network density, there are 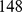 non-cohesive functional units among the total 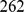 functional units identified by RSRGM. Furthermore, for each PPI network we find most of these revealed non-cohesive functional units are significantly enriched by GO functions. The detailed statistic information of these biological significant non-cohesive units identified by RSRGM for the four PPI networks are presented in Table S3.

**Table 3:** Statistics of functional units identified by RSRGM and the corresponding proteins they cover.

Network	# identified groups	# non-cohesive groups	# GO significant non-cohesive groups
Gavin	177 (1102)	63 (351)	46 (295)
Krogan	262 (1442)	148 (764)	86 (533)
Collins	90 (826)	13 (47)	9 (33)
BioGRID	692 (4771)	451 (3997)	229 (2955)

The numbers in the parentheses are the numbers of proteins covered by identified functional groups.

**Figure 6:**
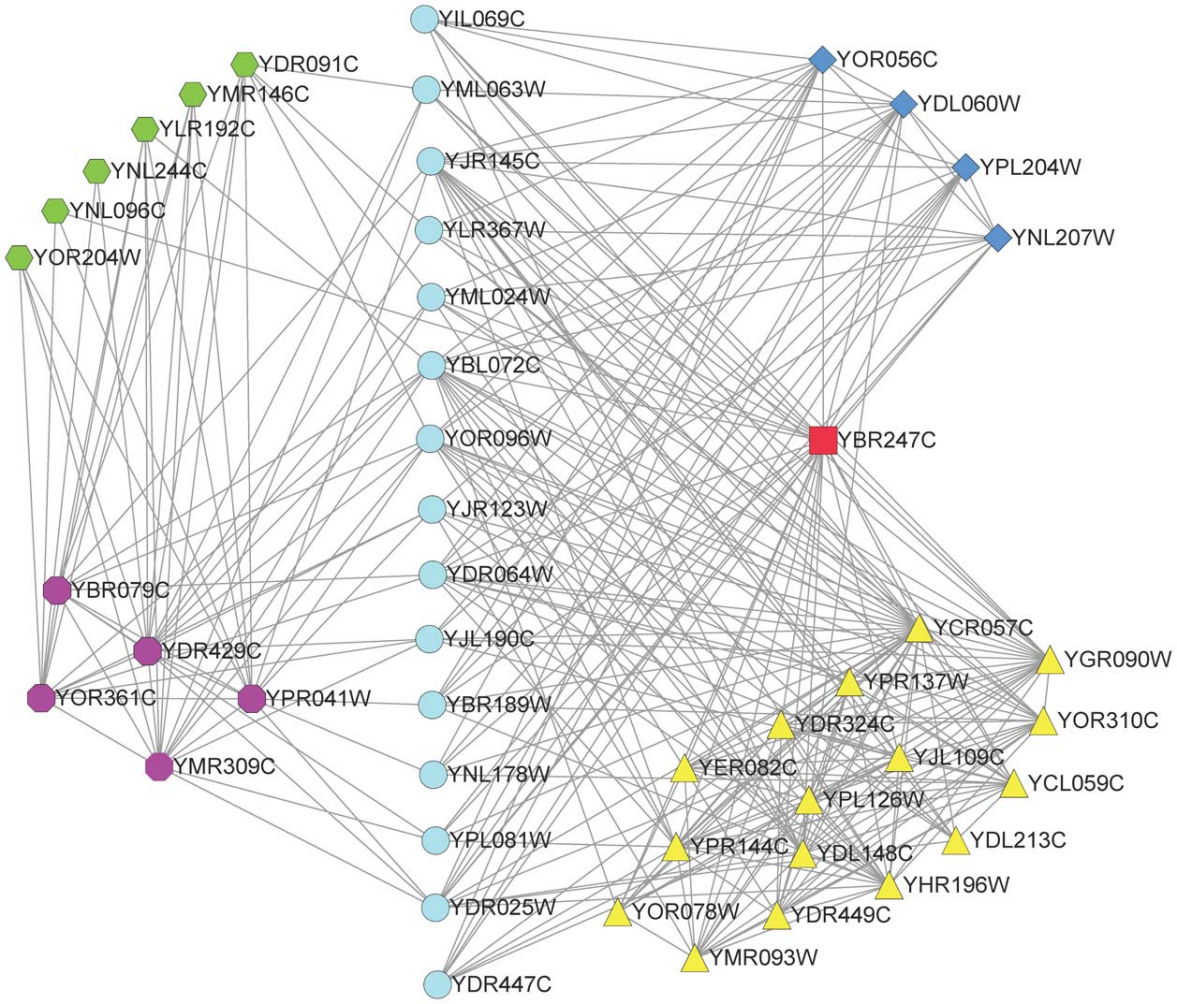
Interactions among detected functional groups 38, 42, 85, 142 and 146 in Gavin network. Proteins are labeled according to groups to which they belong: group 38 (diamond), group 42 (hexagon), group 85 (triangle), group 142 (circle), group 146 (octagon). Protein YBR247C shared by groups 38 and 85 is labeled with rectangle. This figure is plotted with software Cytoscape [45].

Figure 6 shows an example of revealed non-cohesive functional units in Gavin network. Proteins in group 142 seldom interact with each other but have the similar connections to proteins in groups 38, 42, 85 and 146. Group 142 is closely related to biological process of cytoplasmic translation (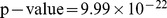), cellular component of cytosolic small ribosomal subunit (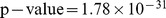) and molecular function of structural constituent of ribosome (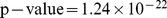). These cytoplasmic translation proteins typically do not interact with other cytoplasmic translation proteins. They often interact with proteins in 43S preribosome (group 38) and proteins in 90S preribosome (group 85). These cytoplasmic translation proteins also have similar interactions with members of groups 42 and 146, both of which are significant enriched in proteins annotated with translational initiation. Interestingly, we find that these translational initiation proteins are grouped into a cohesive functional unit (group 146) and a non-cohesive unit (group 42). This may be because that proteins in group 42 mainly interact with proteins in group 146, but proteins in group 146 not only interact with proteins in the same group but also interact with proteins in group 42. Thus proteins in these two groups have a little different interaction patterns. Anyhow, a method just focusing on cohesive protein complexes would not reveal the biological significant non-cohesive units 142 and 42.

**Figure 7:**
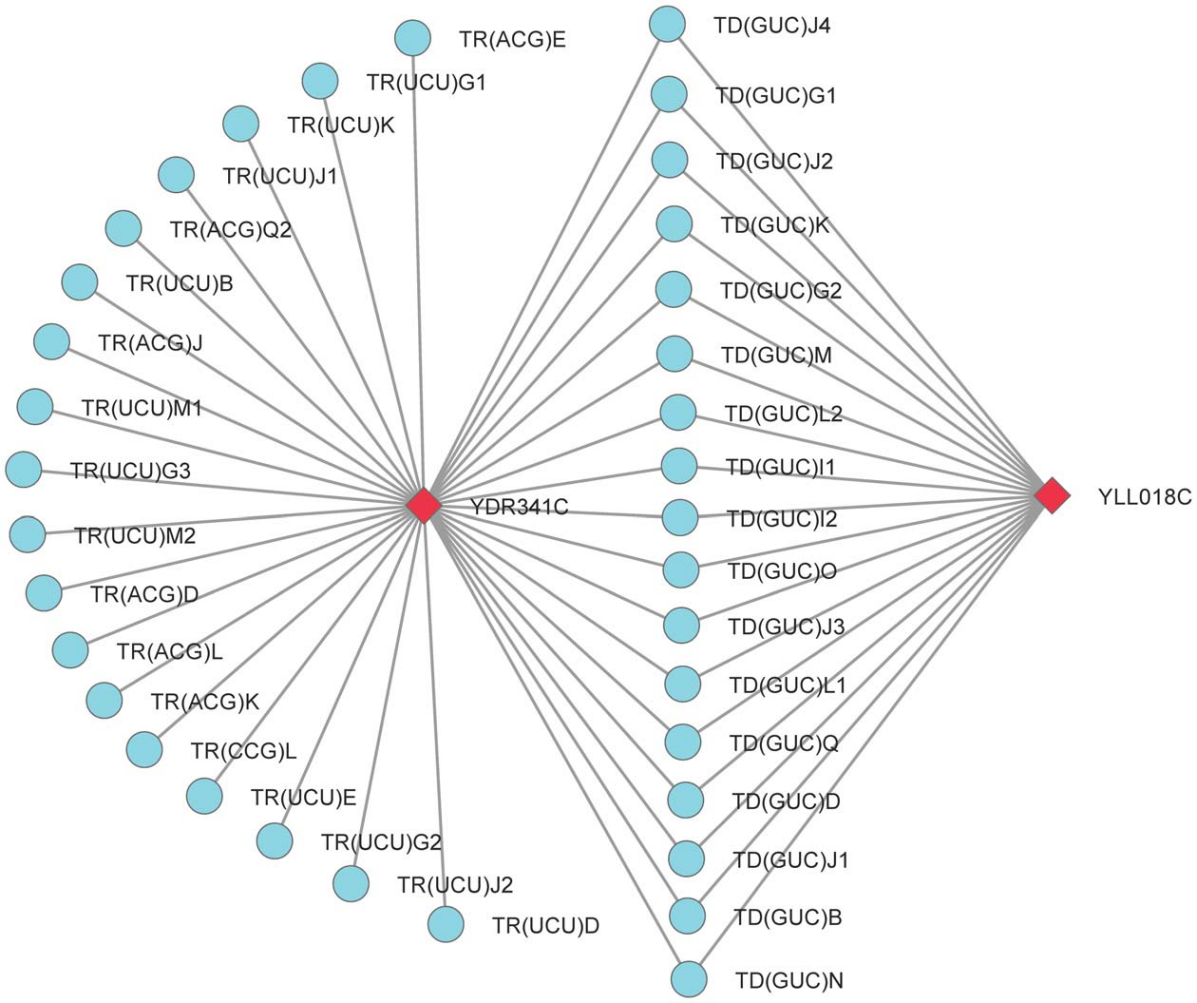
Interactions among detected functional group 431 and proteins YDR341C and YLL018C in BioGRID network. Proteins in group 431 (circle) which consists of spoke proteins are connected by two hub proteins (diamond) YDR341C and YLL018C. This figure is plotted with software Cytoscape [45].

Figure 7 illustrates a spoke functional unit detected by RSRGM in BioGRID network. These spoke proteins in functional group 431 are connected by the two hub proteins YDR341C and YLL018C. Group 431 is significantly enriched in biological process of translational elongation (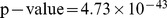), cellular component of cytosol (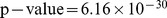) and molecular function of base pairing with mRNA (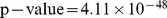). The two hub proteins are annotated with biological processes of aspartyl-tRNA aminoacylation and cytoplasmic translation, cellular component of cytoplasm and molecular function of aspartate-tRNA ligase activity. Thus, it may be that these spoke proteins in group 431, the two hub proteins and some other relative proteins cooperate with each other to finish the process of translation. However, this functional significant spoke model may be overlooked by algorithms which are mainly proposed to detect cohesive protein complexes. More examples of non-cohesive functional units detected by RSRGM for the four PPI networks are presented in Text S4.

## Discussion

Discovering functional units in PPI networks allows for predicting protein function and further unveiling the complex inner working mechanism of cell. Previous algorithms identifying functional units mostly focus on cohesive protein complexes which have more internal interactions than external interactions. However, recent studies have shown that besides cohesive complexes, other structural functional units also exist in PPI networks. Furthermore, traditional algorithms detecting complexes which do not handle overlaps are not always able to present the biological reality. Thus, in this study, we not only concentrate on detecting cohesive complexes, but also pay attention to reveal overlapping and other non-cohesive structural functional units.

A new sparse random graph model is proposed to reveal overlapping and various structural functional units, which is mainly parameterized by two model parameters. Different from conventional definition of cohesive protein complexes, we use one parameter to define functional units as groups of proteins which have the similar connections to others. Thus functional units with various topological structure can be revealed. Different from traditional hard clustering, we use the other parameter to present the degree of proteins belonging to groups. Thus our model allows a protein to be shared by more than one identified functional unit. The sparse priors given to these two parameters not only have good biological interpretation but also help to determinate the value of possible number of functional units in PPI networks. Experimental results on four yeast PPI networks show that our model performs well not only on detecting cohesive protein complexes and overlapping complexes, but also on revealing other non-cohesive functional units.

In fact, two other algorithms [21], [22] have been developed to mine the non-cohesive functional units in PPI networks. We do not compare RSRGM to Pinkert method [21] not only for it mainly focuses on functional units on large scale (here we focus on small scale at protein complex level) but also for there is no public software available. And we also do not present the comparative results of BTS [22] because it can not analyze the four PPI networks except Collins in 48 hours. Hence, we mainly compare our model to algorithms developed to identify protein complexes and perform functional homogeneity analysis of non-cohesive functional units revealed by RSRGM. Anyhow, our analysis shows that PPI networks are more than sparsely interacting protein complexes. Rather, functional units beyond cohesive complexes also widely exist.

Even though random graph models have been used to capture the properties and evolution mechanism of PPI network, and several recent studies have explored their new applications in analyzing PPI networks, the technique presented in this study is one of the first to use random graph models for the purpose of mining functional units. Different from traditional approaches detecting cohesive protein complexes based on dense subnetwork detection or graph partition, our approach gives new insights about the workability of random graph models in exploring functional structure of PPI networks.

Our method can be extended in the following aspects. First, we use Poisson distribution to generate connections among proteins for its simplicity. Other distributions (e.g., Bernoulli distribution and binomial distribution) can also be tried to find which one is more appropriate for the task at hand. Second, in this study, we determinate values of the two key parameters 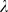 and 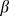 by the trick of grid searching, which is more time-consuming. Although we experimentally find that the values of 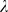 and 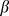 that belong to 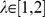 and 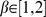 usually lead to reasonable results, it also needs to take an effective and efficient measure to determine the values of these two parameters. Third, our random graph model is mainly devised for PPI networks. It is interesting to extend and apply it to explore structure in other biological networks such as gene regulatory and cell signaling networks.

## Supporting Information

Table S1 (XLS)

Table S2 (XLS)

Table S3 (XLS)

Text S1 **Solution to regularized sparse random graph model.**
(PDF)

Text S2 **Metrics for evaluating protein complexes detection.**
(PDF)

Text S3 **Parameter settings of compared algorithms.**
(PDF)

Text S4 **Examples of non-cohesive functional units detected by RSRGM.**
(PDF)
