# Supplementary material for: Exploring Overlapping Functional Units with Various Structure in Protein Interaction Networks
Source: PLoS One. 2012 Aug 20;7(8):e43092. doi: 10.1371/journal.pone.0043092 (PMC3423443; doi:10.1371/journal.pone.0043092)
Supplement: Text S1 — Solution to regularized sparse random graph model. (PDF) [file pone.0043092.s004.pdf]

Solution to regularized sparse random graph model

The objective function of the regularized sparse random graph model (RSRGM) is:

$$\left\{\begin{array}{lc} \min_{\theta,\psi} & -\sum_{i,j=1}^{n} A_{ij}\log\left((\theta\psi)_{ij}\right)+\sum_{i,j=1}^{n}(\theta\psi)_{ij} \\ & +\lambda\sum_{i=1}^{n}\sum_{k=1}^{K}\theta_{ik}+\lambda\sum_{k=1}^{K}\sum_{j=1}^{n}\psi_{kj} \\ & +\frac{\beta}{2}\left(Tr\left(\theta^{T}D\theta\right)+Tr\left(\psi D\psi^{T}\right)-2Tr\left(\psi A\theta\right)\right) \\ s.t. & \theta\geq 0,\psi\geq 0. \end{array}\right. \tag{1}$$

Here, we use the multiplicative updating rule [2] to solve this nonnegative constrained optimization problem.

Let ϕ_{ik} and ω_{kj} be the Lagrange multipliers for constraints $\theta_{ik}\geq 0$ and $\psi_{kj}\geq 0$, respectively, and $\phi=(\phi_{ik})$, $\omega=(\omega_{kj})$. The Lagrange function $\mathcal{L}$ is

$$\begin{aligned} \mathcal{L}\left(\theta,\psi,\phi,\omega\right) = & -\sum_{i,j=1}^{n} A_{ij}\log\left((\theta\psi)_{ij}\right)+\sum_{i,j=1}^{n}(\theta\psi)_{ij}+\lambda\sum_{i=1}^{n}\sum_{k=1}^{K}\theta_{ik}+\lambda\sum_{k=1}^{K}\sum_{j=1}^{n}\psi_{kj} \\ & +\frac{\beta}{2}\left(Tr\left(\theta^{T}D\theta\right)+Tr\left(\psi D\psi^{T}\right)-2Tr\left(\psi A\theta\right)\right) \\ & +\sum_{i=1}^{n}\sum_{k=1}^{K}\phi_{ik}\theta_{ik}+\sum_{k=1}^{K}\sum_{j=1}^{n}\omega_{kj}\psi_{kj}. \end{aligned} \tag{2}$$

The gradients of Lagrange function $\mathcal{L}$ with respect to θ_{ik} and ψ_{kj} are:

$$\nabla_{\theta_{ik}}\mathcal{L}=-\sum_{j=1}^{n}A_{ij}\frac{\psi_{kj}}{(\theta\psi)_{ij}}+\sum_{j=1}^{n}\psi_{kj}+\lambda+\beta(D\theta)_{ik}-\beta\left(A\psi^{T}\right)_{ik}+\phi_{ik}, \tag{3}$$

and

$$\nabla_{\psi_{kj}}\mathcal{L}=-\sum_{i=1}^{n}A_{ij}\frac{\theta_{ik}}{(\theta\psi)_{ij}}+\sum_{i=1}^{n}\theta_{ik}+\lambda+\beta(\psi D)_{kj}-\beta\left(\theta^{T}A\right)_{kj}+\omega_{kj}. \tag{4}$$

Since the estimators of θ_{ik} and ψ_{kj} need to satisfy $\nabla_{\theta_{ik}}\mathcal{L} = 0$ and $\nabla_{\psi_{kj}}\mathcal{L} = 0$, we can get

$$\phi_{ik} = \sum_{j=1}^{n} A_{ij}\frac{\psi_{kj}}{(\theta\psi)_{ij}} - \sum_{j=1}^{n}\psi_{kj} - \lambda - \beta(D\theta)_{ik} + \beta\left(A\psi^{T}\right)_{ik}, \tag{5}$$

and

$$\omega_{kj} = \sum_{i=1}^{n} A_{ij}\frac{\theta_{ik}}{(\theta\psi)_{ij}} - \sum_{i=1}^{n}\theta_{ik} - \lambda - \beta(\psi D)_{kj} + \beta\left(\theta^{T}A\right)_{kj}. \tag{6}$$

Using the Karush-Kuhn-Tucker (KKT) conditions [1], $\phi_{ik}\theta_{ik} = 0$ and $\omega_{kj}\psi_{kj} = 0$, we get the following equations for θ_{ik} and ψ_{kj}:

$$\theta_{ik}\left(\sum_{j=1}^{n}\psi_{kj} + \lambda + \beta(D\theta)_{ik}\right) = \theta_{ik}\left(\sum_{j=1}^{n} A_{ij}\frac{\psi_{kj}}{(\theta\psi)_{ij}} + \beta\left(A\psi^{T}\right)_{ik}\right), \tag{7}$$

and

$$\psi_{kj}\left(\sum_{i=1}^{n}\theta_{ik} + \lambda + \beta(\psi D)_{kj}\right) = \psi_{kj}\left(\sum_{i=1}^{n} A_{ij}\frac{\theta_{ik}}{(\theta\psi)_{ij}} + \beta\left(\theta^{T}A\right)_{kj}\right). \tag{8}$$

Then, it is easy to obtain the updating rules:

$$\theta_{ik} \leftarrow \theta_{ik}\frac{\sum_{j=1}^{n} A_{ij}\frac{\psi_{kj}}{(\theta\psi)_{ij}} + \beta\left(A\psi^{T}\right)_{ik}}{\sum_{j=1}^{n}\psi_{kj} + \lambda + \beta(D\theta)_{ik}}, \tag{9}$$

and

$$\psi_{kj} \leftarrow \psi_{kj}\frac{\sum_{i=1}^{n} A_{ij}\frac{\theta_{ik}}{(\theta\psi)_{ij}} + \beta\left(\theta^{T}A\right)_{kj}}{\sum_{i=1}^{n}\theta_{ik} + \lambda + \beta(\psi D)_{kj}}. \tag{10}$$

By initializing θ and ψ randomly and updating θ and ψ alternatively according to Equations (9) and (10), respectively, we can obtain the solution to the regularized sparse random graph model (1).

References

[1] H.W. Kuhn and A.W. Tucker. Nonlinear programming. In *Proceedings of the second Berkeley symposium on mathematical statistics and probability*, volume 1, pages 481–492. California, 1951.

[2] Daniel D. Lee and H. Sebastian Seung. Algorithms for non-negative matrix factorization. In *Advances in neural information processing systems*, volume 13, pages 556–562, 2001.
