# Supplementary material for: Exploring Overlapping Functional Units with Various Structure in Protein Interaction Networks
Source: PLoS One. 2012 Aug 20;7(8):e43092. doi: 10.1371/journal.pone.0043092 (PMC3423443; doi:10.1371/journal.pone.0043092)
Supplement: Text S2 — Metrics for evaluating protein complexes detection. (PDF) [file pone.0043092.s005.pdf]

Metrics for evaluating protein complexes detection

We utilize three measures for evaluating performance of algorithms based on overlaps between the identified complexes and reference complexes. The first one is accuracy (Acc) which is defined by Brohée and Van Helden [1] and the other two measures we use are Jaccard and PR introduced by Song and Singh [2]. Before describing these metrics, we introduce some notations first. Let M be the number of complexes detected by a particular computational algorithm, and N be the number of reference complexes against which we are evaluating. C_j denotes the set of proteins that belong to detected complex j, and G_i represents the set of proteins associated with the true complex i.

Acc: let $T_{ij} = |G_i \cap C_j|$ denotes the number of proteins shared between reference complex i and detected complex j. Then the sensitivity (Sn) and positive predictive value (PPV) are defined as follows:

$$\text{Sn} = \frac{\sum_{i=1}^{N} \max_j T_{ij}}{\sum_{i=1}^{N} |G_i|} \qquad \text{and} \qquad \text{PPV} = \frac{\sum_{j=1}^{M} \max_i T_{ij}}{\sum_{j=1}^{M} |(\cup_{i=1}^{N} G_i) \cap C_j|}.$$

Note that here we use the new definition of PPV introduced by Xie *et al.* [3] which is a little bit different from the original definition, since the new definition is more suitable for evaluating overlapping clusters. Then Acc is defined as the geometric average of sensitivity and PPV:

$$\text{Acc} = \sqrt{\text{Sn} \times \text{PPV}}.$$

Jaccard: let $Jac_{ij} = \frac{|G_i \cap C_j|}{|G_i \cup C_j|}$ denotes Jaccard coefficient between reference complex i and detected complex j. The Jaccard measure for real complex i is defined as $JaccardG_i = \max_j Jac_{i,j}$. Taking an average over these reference complexes, weighted by complex size, we get an overall score for the N reference

complexes:

$$JaccardG = \frac{\sum_{i=1}^{N} |G_i| JaccardG_i}{\sum_{i=1}^{N} |G_i|}.$$

Similarly, the Jaccard measure for detected complex j is defined as $JaccardC_j = \max_i Jac_{i,j}$, and overall score for the M detected complexes is defined as follows:

$$JaccardC = \frac{\sum_{j=1}^{M} |C_j| JaccardC_j}{\sum_{j=1}^{M} |C_j|}.$$

Finally, Jaccard between the M detected complexes and N reference complexes is defined as the harmonic mean of $JaccardC$ and $JaccardG$:

$$\text{Jaccard} = \frac{2 \times JaccardC \times JaccardG}{JaccardC + JaccardG}.$$

PR: let $PR_{ij} = \frac{|G_i \cap C_j|}{|G_i|} \times \frac{|G_i \cap C_j|}{|C_j|}$ denotes the precision-recall (PR)-based score between reference complex i and detected complex j. The first part $\frac{|G_i \cap C_j|}{|G_i|}$ represents how much of reference complex i is recovered by detected complex j, and the second part $\frac{|G_i \cap C_j|}{|C_j|}$ quantify what fraction of proteins in detected complex j corresponding to real complex i. Similar to the definition of Jaccard, we also define $PRG_i = \max_j PR_{ij}$, $PRC_j = \max_i PR_{ij}$, $PRG = \frac{\sum_{i=1}^{N} |G_i| PRG_i}{\sum_{i=1}^{N} |G_i|}$ and $PRC = \frac{\sum_{j=1}^{M} |C_j| PRC_j}{\sum_{j=1}^{M} |C_j|}$. Then the overall PR score between detected complexes and real complexes is defined as the harmonic mean of PRG and PRC:

$$\text{PR} = \frac{2 \times PRG \times PRC}{PRG + PRC}.$$

References

[1] S. Brohée and J. Van Helden. Evaluation of clustering algorithms for protein-protein interaction networks. *BMC Bioinformatics*, 7(1):488, 2006.

[2] J. Song and M. Singh. How and when should interactome-derived clusters be used to predict functional modules and protein function? *Bioinformatics*, 25(23):3143–3150, 2009.

[3] Z. Xie, C.K. Kwoh, X.L. Li, and M. Wu. Construction of co-complex score matrix for protein complex prediction from ap-ms data. *Bioinformatics*, 27(13):i159–i166, 2011.
