# Supplementary material for: Exploring Overlapping Functional Units with Various Structure in Protein Interaction Networks
Source: PLoS One. 2012 Aug 20;7(8):e43092. doi: 10.1371/journal.pone.0043092 (PMC3423443; doi:10.1371/journal.pone.0043092)
Supplement: Text S3 — Parameter settings of compared algorithms. (PDF) [file pone.0043092.s006.pdf]

Parameter settings of compared algorithms

To test the effectiveness of RSRGM in detecting protein complexes, we compare the performance of RSRGM to a representative set of previous competitive algorithms: CFinder [1], ClusterOne [6], CMC [5], MCL [3], MCODE [2], MINE [8] and SPICi [4]. Table 1 lists the websites where we download the softwares of corresponding algorithms, the version numbers of the softwares we have used to test them and indications whether these algorithms support for detecting overlapping complexes. Before presenting the detailed parameter settings for each algorithm, we declare several general considerations first. Different from classification algorithms which can divide the data into a training set and a testing set and then tune parameters on the training set, the algorithms considered in this study correspond to clustering algorithms in machine learning which can not determine parameters similarly to classification algorithms. Thus, for each algorithm, we optimize the parameters that yield best results. In order to avoid evaluation bias, we decide on the following:

- We use three quality metrics to evaluate the performance of each algorithms: Acc, Jaccard and PR.
- We use two different gold standards: the MIPS complexes and the SGD complexes.
- For each algorithm, similar to RSRGM, the final results are obtained after optimizing the algorithm parameters to yield best possible results as measured in terms of harmonic mean of six scores (Acc on MIPS, Jaccard on MIPS, PR on MIPS, Acc on SGD, Jaccard on SGD, and PR on SGD).

CFinder

CFinder [1] is one of the first algorithms that is designed to detect overlapping modules in biological networks based on Clique Percolation Method [7]. A key

Table 1: Characteristics of algorithms compared in this paper

Algorithm	Downloading website	Version	Overlapping
CFinder	http://cfinder.org/	2.0.5	√
ClusterONE	http://www.paccanarolab.org/cluster-one/index.html	0.94	√
CMC	http://www.comp.nus.edu.sg/~wongls/projects/complexprediction/CMC-26may09/	2.0	√
MCL	http://micans.org/mcl/	09-308	
MCODE	http://baderlab.org/Software/MCODE	1.32	√
MINE	http://www.biomedcentral.com/1471-2105/12/192	1.5	√
SPICi	http://compbio.cs.princeton.edu/spici/	Unknown (24 April 2012)	

Table 2: Parameters selected for CFinder

Network	Gavin	Krogan	Collins	BioGRID
k-clique size	5	5	8	N/A

N/A for BioGRID network indicates that CFinder can not give any results within 48 hours.

parameter of CFinder is the k-clique size. In this study, for each PPI network, we test CFinder with k-clique size from 3 to 10. Table 2 lists the optimal values of parameter k on the four PPI networks.

ClusterONE

ClusterONE [6] is a new algorithm detecting overlapping protein complexes in PPI networks based on overlapping neighborhood expansion. As suggested by the authors, we do not tune the parameters to a particular network and for all the four PPI networks we use the default settings of parameters in the software.

CMC

CMC [5] detects overlapping protein complexes by first finding maximal cliques in PPI networks, and then removing or merging highly overlapped maximal cliques based on their connectivity. Thus CMC is primarily governed by the overlap threshold and merging threshold. In this study, the overlap threshold is tested between 0.2 and 0.8 with a step size of 0.1, the merging threshold is tested on uniformly sampled real values between 0 and 1 with a step size of 0.1. And the minimum size of detected complexes is set to be 3. Table 3 lists the optimal overlap threshold and merging threshold for the four PPI networks.

Table 3: Parameters selected for CMC

Network	Gavin	Krogan	Collins	BioGRID
Overlap threshold	0.6	0.2	0.6	0.2
Merging threshold	0.5	0.5	0.3	0.9

Table 4: Parameters selected for MCL

Network	Gavin	Krogan	Collins	BioGRID
Inflation	3.2	1.8	2.8	3.4

MCL

MCL [3] is a competing protein complexes detection algorithm based on Markov clustering. The key parameter of MCL is inflation, which tunes the granularity of clustering. Here, we try inflation values from 1.2 to 5.0 with 0.2 increment. And we list the optimal inflation for the four PPI networks in Table 4.

MCODE

MCODE [2] is one of the first protein complexes discovery algorithms, which consists of three phases: vertex weighting, protein complex formation and post-processing. The depth limit parameter controls how far the growth process is willing to proceed from the seed protein when considering other proteins to be added to the seed protein to form a protein complex. The node score cutoff is the most influence parameter for complex size which controls how much difference is allowed between scores of proteins within the same complex. There are two possible post-processing operations: haircut and fluffing. Note that MCODE is able to produce overlapping complexes in the fluffing phase, but we experimentally find that it performs better when fluffing is turned off. We try all the possible combinations of the following parameters:

- Depth limit: 3, 4, 5
- Node score cutoff: 0.1 to 1.0 with a step size of 0.1
- Haircut: on or off
- Fluffing: on or off

Table 5: Parameters selected for MCODE

Network	Gavin	Krogan	Collins	BioGRID
Depth limit	3	3	3	3
Node score cutoff	0.4	0.5	0.2	0.1
Haircut	on	on	on	on
Fluffing	off	off	off	off
Node density cutoff	N/A	N/A	N/A	N/A

Table 6: Parameters selected for MINE

Network	Gavin	Krogan	Collins	BioGRID
Depth limit	3	3	3	3
Node score cutoff	0.1	0.1	0.1	0.1

- Node density cutoff: 0, 0.1, 0.2

We list the optimal parameters of MCODE for the four PPI networks in Table 5.

MINE

MINE [8] identifies highly modular sets of proteins within highly interconnected PPI networks. In this study, we test it over a range of 10 settings of node score cutoff (0.1-1) and 3 settings of depth limit (3, 4, 5). For the other parameters without stating, we use the default values in the software. The optimal parameters of MINE for the four PPI networks are listed in Table 6.

SPICi

SPICi [4] is a fast local network clustering algorithm for large biological networks, which can be naturally used to detect protein complexes from PPI networks. Here, we test density threshold from 0.1 to 1 with 0.1 increment. Table 7 lists the optimal values of density parameter for the four PPI networks.

Table 7: Parameters selected for SPICi

Network	Gavin	Krogan	Collins	BioGRID
Density	0.7	0.7	0.7	0.8

References

[1] B. Adamcsek, G. Palla, I.J. Farkas, I. Derényi, and T. Vicsek. Cfinder: locating cliques and overlapping modules in biological networks. *Bioinformatics*, 22(8):1021 – 1023, 2006.

[2] Gary D Bader and Christopher WV Hogue. An automated method for finding molecular complexes in large protein interaction networks. *BMC Bioinformatics*, 4(1):2, 2003.

[3] A.J. Enright, S. Van Dongen, and C.A. Ouzounis. An efficient algorithm for large-scale detection of protein families. *Nucleic Acids Research*, 30(7):1575–1584, 2002.

[4] Peng Jiang and Mona Singh. Spici: a fast clustering algorithm for large biological networks. *Bioinformatics*, 26(8):1105 – 1111, 2010.

[5] G. Liu, L. Wong, and H.N. Chua. Complex discovery from weighted ppi networks. *Bioinformatics*, 25(15):1891 – 1897, 2009.

[6] T. Nepusz, H. Yu, and A. Paccanaro. Detecting overlapping protein complexes in protein-protein interaction networks. *Nature Methods*, 9(5):471–472, 2012.

[7] Gergely Palla, Imre Derenyi, Illes Farkas, and Tamas Vicsek. Uncovering the overlapping community structure of complex networks in nature and society. *Nature*, 435(7043):814–818, 2005.

[8] Kahn Rhrissorrakrai and Kristin C Gunsalus. Mine: module identification in networks. *BMC Bioinformatics*, 12(1):192, 2011.
