# Supplementary material for: Exploring Overlapping Functional Units with Various Structure in Protein Interaction Networks
Source: PLoS One. 2012 Aug 20;7(8):e43092. doi: 10.1371/journal.pone.0043092 (PMC3423443; doi:10.1371/journal.pone.0043092)
Supplement: Text S4 — Examples of non-cohesive functional units detected by RSRGM. (PDF) [file pone.0043092.s007.pdf]

Examples of non-cohesive functional units detected by RSRGM

References

[1] M.S. Cline, M. Smoot, E. Cerami, A. Kuchinsky, N. Landys, C. Workman, R. Christmas, I. Avila-Campilo, M. Creech, B. Gross, Kristina Hanspers, Ruth Isserlin, Ryan Kelley, Sarah Killcoyne, Samad Lotia, Steven Maere, John Morris, Keiichiro Ono, Vuk Pavlovic, Alexander R Pico, Aditya Vailaya, Peng-Liang Wang, Annette Adler, Bruce R Conklin, Leroy Hood, Martin Kuiper, Chris Sander, Ilya Schmulevich, Benno Schwikowski, Guy J Warner, Trey Ideker, and Gary D Bader. Integration of biological networks and gene expression data using cytoscape. *Nature Protocols*, 2(10):2366–2382, 2007.

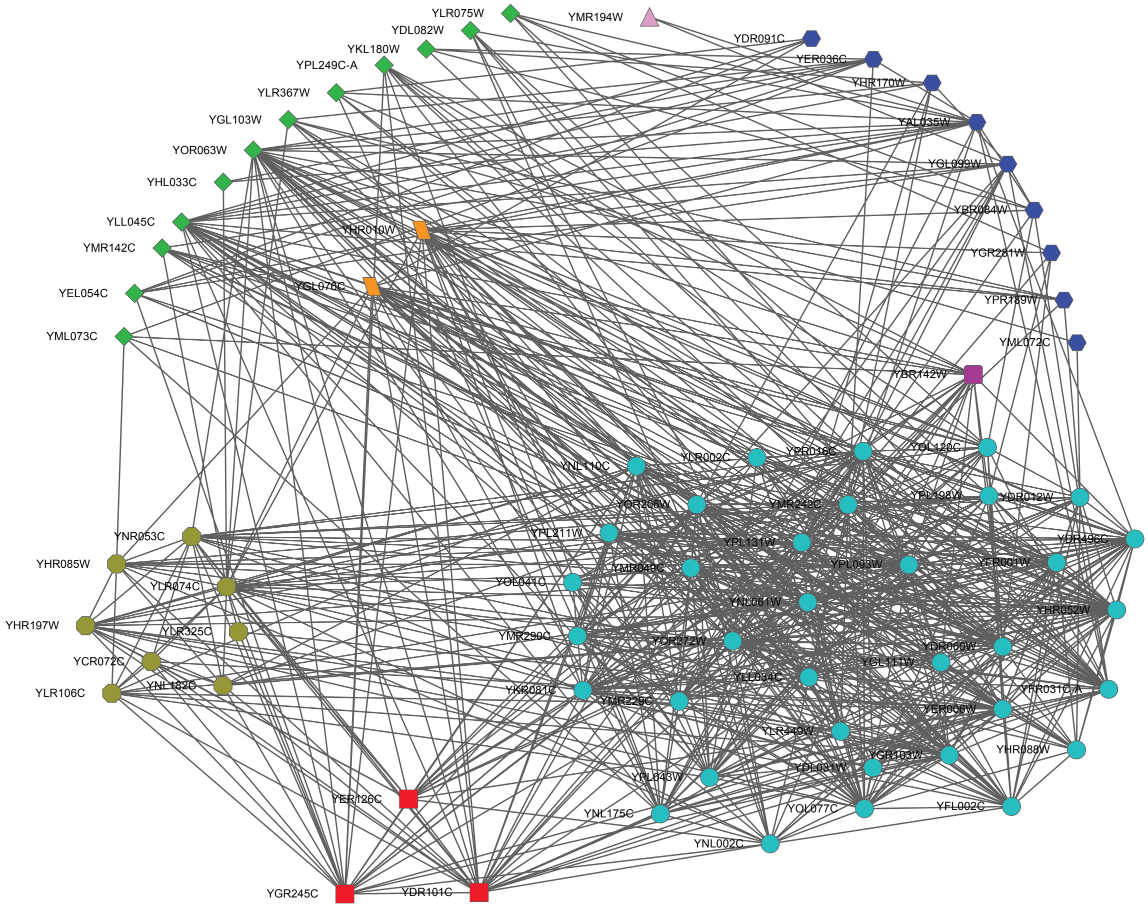

Figure 1: Interactions among functional groups 12, 25, 35 and 79 detected by RSRGM in Gavin network. Proteins are labeled according to group to which they belong: group 12 (circle), group 25 (octagon), group 35 (diamond), group 79 (hexagon). Proteins YDR101C, YGR245C and YER126C shared by groups 12 and 25 are labeled with rectangle; proteins YHR010W and YGL076C shared by groups 12 and 35 are labeled with parallelogram; protein YBR142W shared by groups 12 and 79 is labeled with round rectangle; protein YMR194W shared by groups 35 and 79 is labeled with triangle. Groups 35 and 79 are two identified non-cohesive functional units. This figure is plotted with software Cytoscape [1].

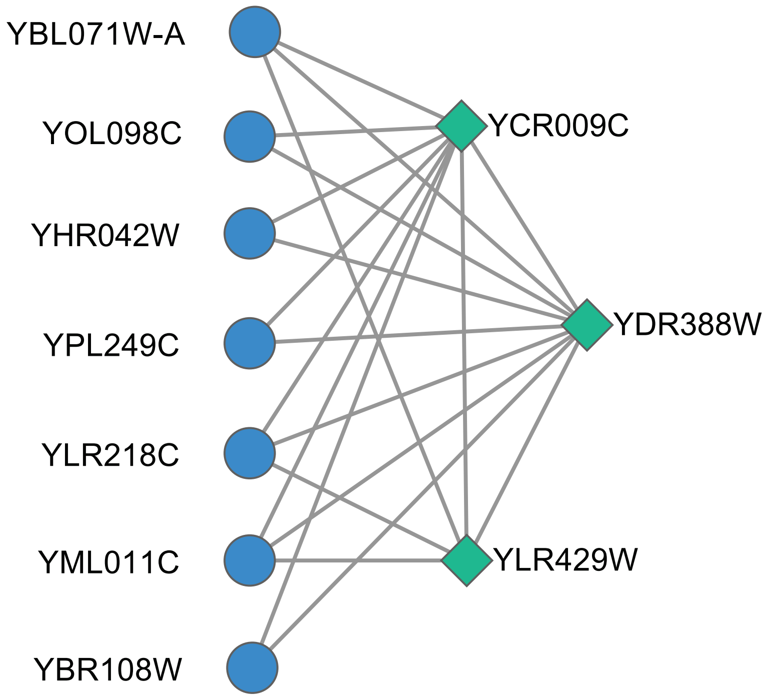

Figure 2: Interactions among functional groups 9 and 181 detected by RSRGM in Krogan network. Proteins are labeled according to group to which they belong: group 9 (circle), group 181 (diamond). Group 9 is an identified non-cohesive functional unit. This figure is plotted with software Cytoscape [1].

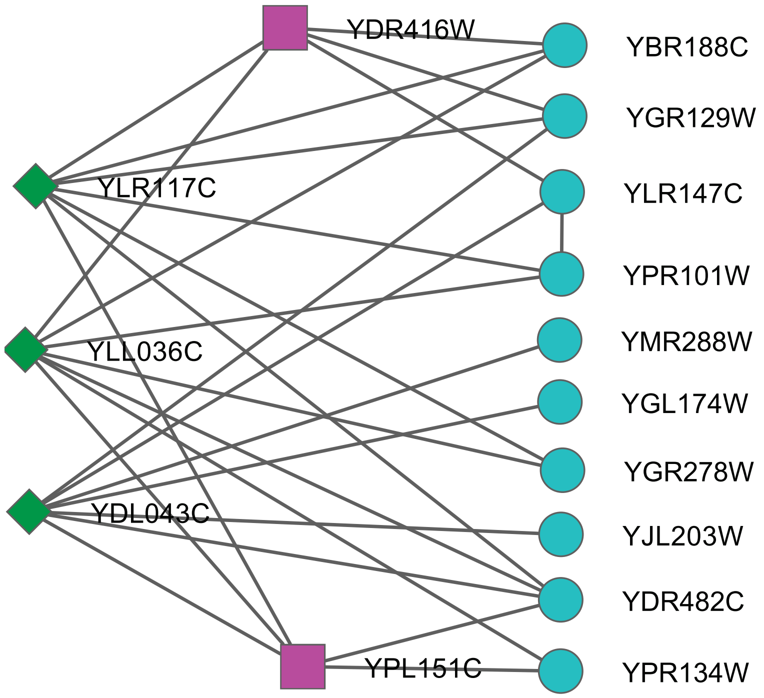

Figure 3: Interactions among functional groups 113 and 129 detected by RSRGM in Krogan network. Proteins are labeled according to group to which they belong: group 113 (diamond), group 129 (circle). Proteins YDR416W and YPL151C shared by these two groups are labeled with rectangle. These two groups are identified non-cohesive functional units. This figure is plotted with software Cytoscape [1].

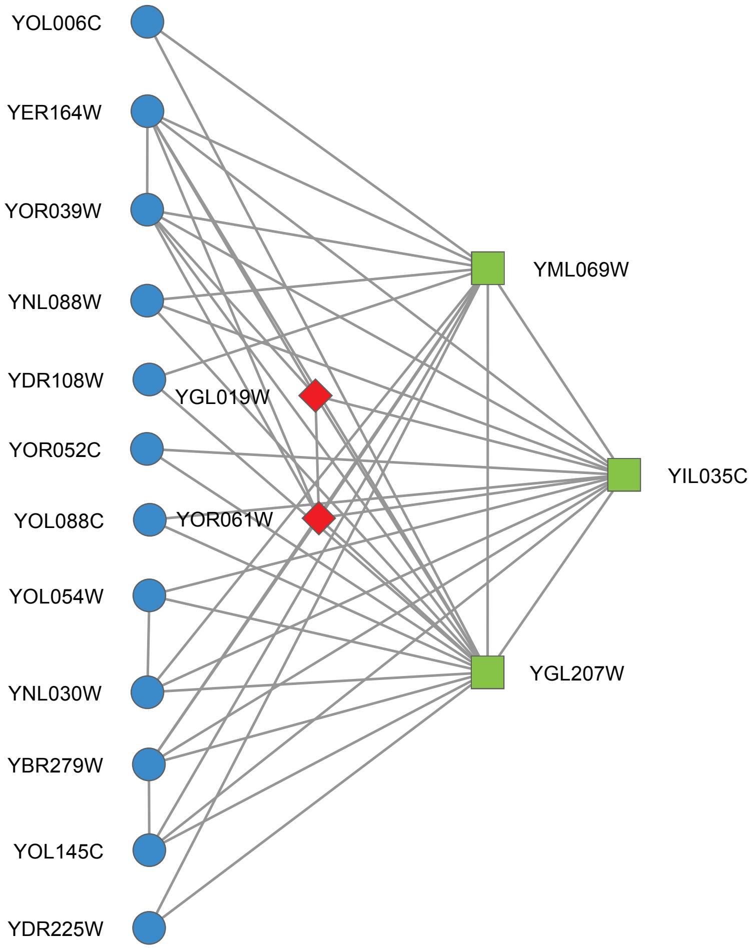

Figure 4: Interactions among functional groups 148 and 149 detected by RSRGM in Krogan network. Proteins are labeled according to group to which they belong: group 148 (circle), group 149 (rectangle). Proteins YGL019W and YOR061W shared by these two groups are labeled with diamond. Group 148 is an identified non-cohesive functional unit. This figure is plotted with software Cytoscape [1].

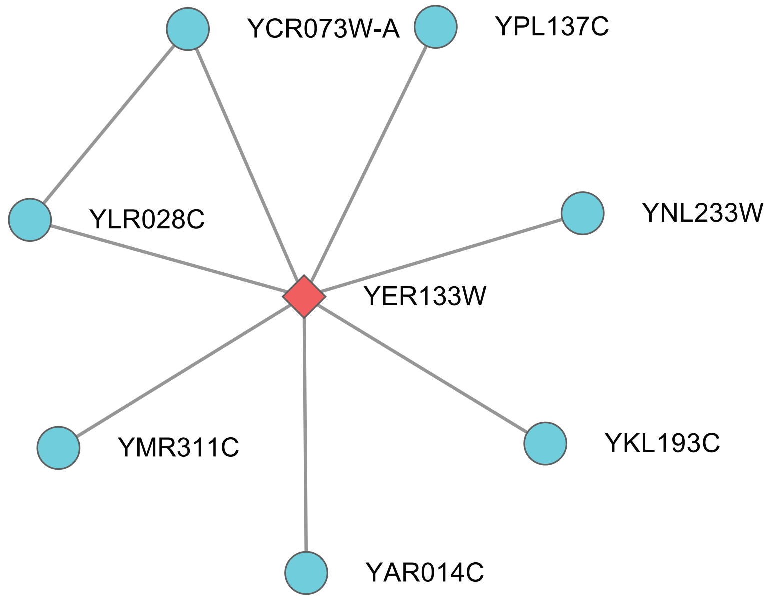

Figure 5: Interactions among functional group 75 detected by RSRGM and hub protein YER133W in Collins network. Proteins belonging to group 75 are labeled with circle and hub protein are labeled with diamond. Group 75 is an identified non-cohesive functional unit. This figure is plotted with software Cytoscape [1].

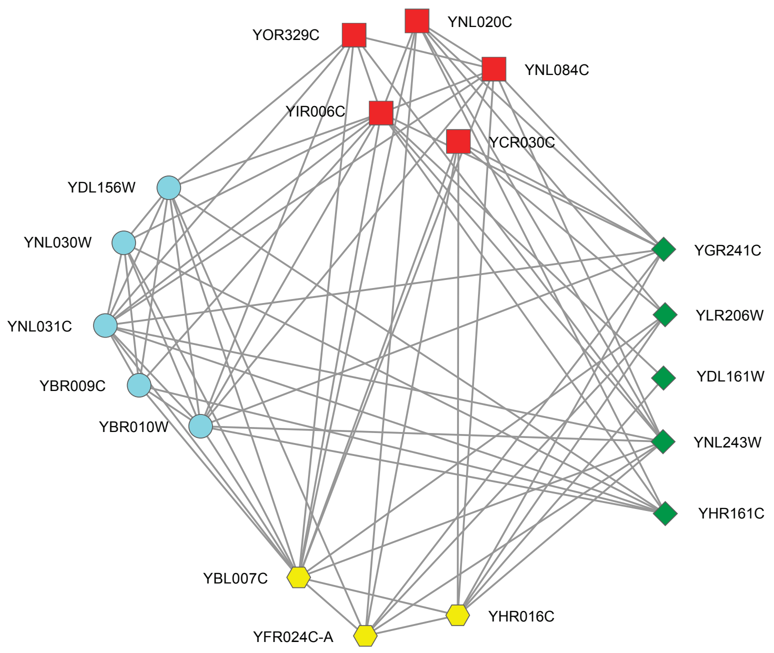

Figure 6: Interactions among functional groups 25, 30, 476 and 573 detected by RSRGM in BioGRID network. Proteins are labeled according to group to which they belong: group 25 (circle), group 30 (diamond), group 476 (rectangle), group 573 (hexagon). Group 30 is an identified non-cohesive functional unit. This figure is plotted with software Cytoscape [1].

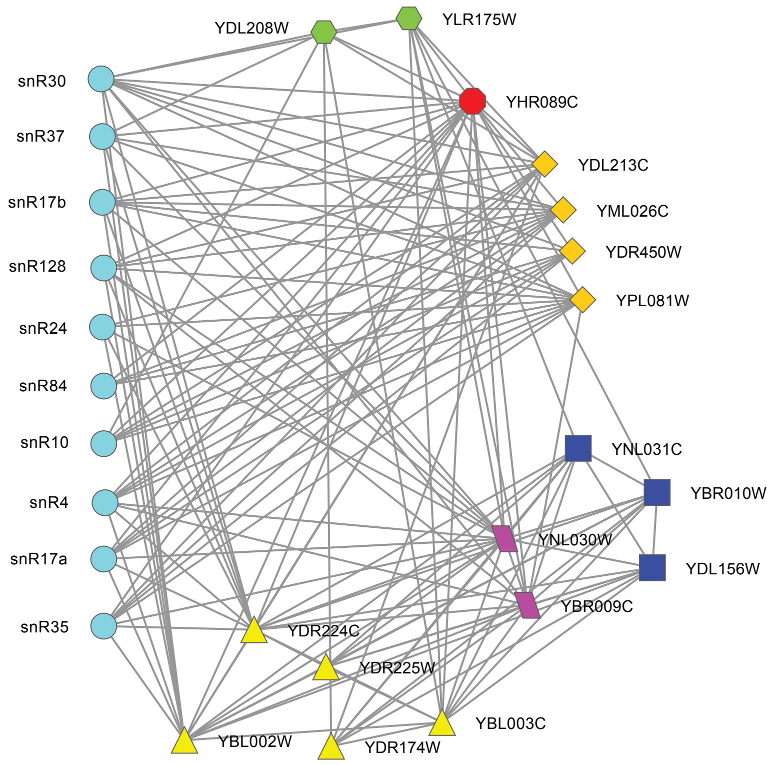

Figure 7: Interactions among functional groups 25, 183, 380, 530 and 659 detected by RSRGM in BioGRID network. Proteins are labeled according to group to which they belong: group 25 (rectangle), group 183 (circle), group 380 (hexagon), group 530 (diamond), group 659 (triangle). Protein YHR089C shared by groups 380 and 530 is labeled with octagon, and proteins YNL030W and YBR009C shared by groups 25 and 659 are labeled with parallelogram. Groups 183 and 530 are two identified non-cohesive functional units. This figure is plotted with software Cytoscape [1].

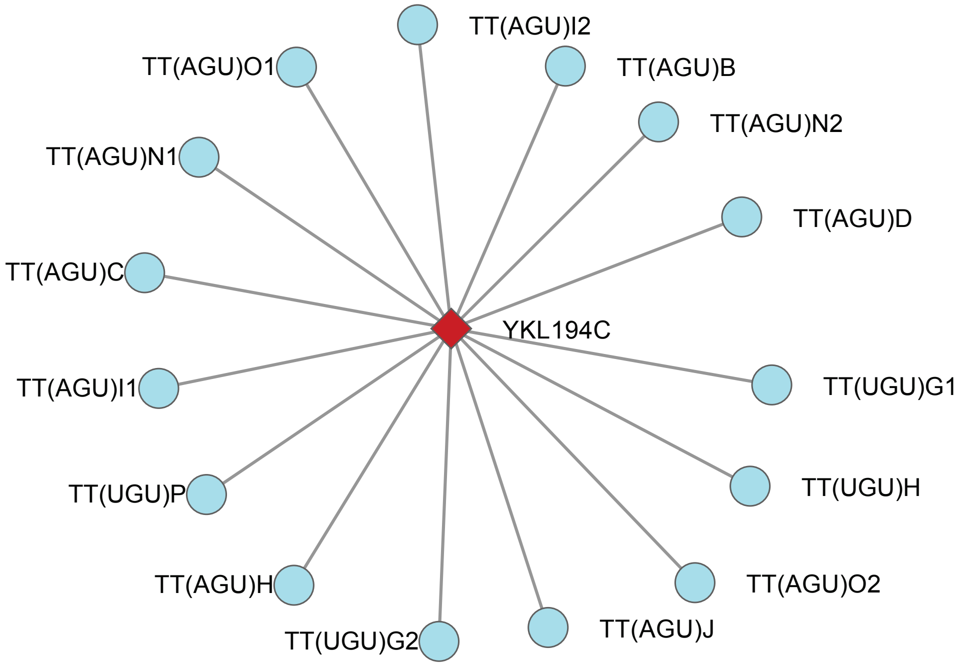

Figure 8: Interactions among functional group 113 detected by RSRGM and hub protein YKL194C in BioGRID network. Proteins belonging to functional group 113 are labeled with circle and hub protein YKL194C is labeled with diamond. Group 113 is an identified non-cohesive functional unit. This figure is plotted with software Cytoscape [1].

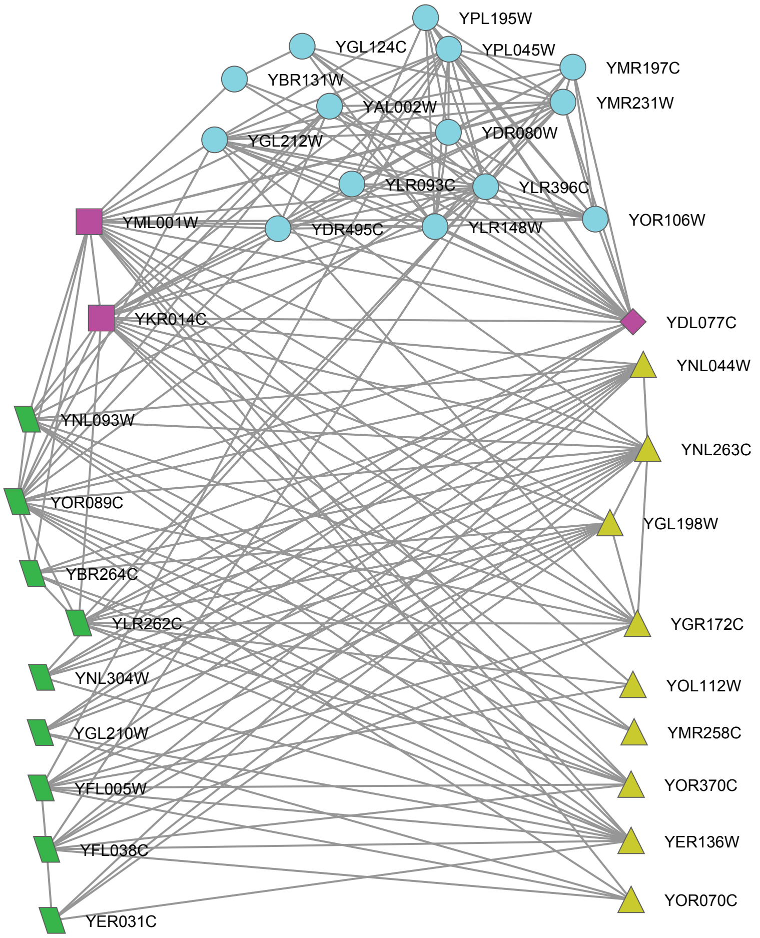

Figure 9: Interactions among functional groups 37, 115 and 451 detected by RSRGM in BioGRID network. Proteins are labeled according to group to which they belong: group 37 (parallelogram), group 115 (triangle), group 451 (circle). Proteins YKR014C and YML001W share by groups 37 and 451 are labeled with rectangle, and protein YDL077C shared by groups 115 and 451 are labeled with diamond. Groups 37 and 115 are two identified non-cohesive functional units. This figure is plotted with software Cytoscape [1].

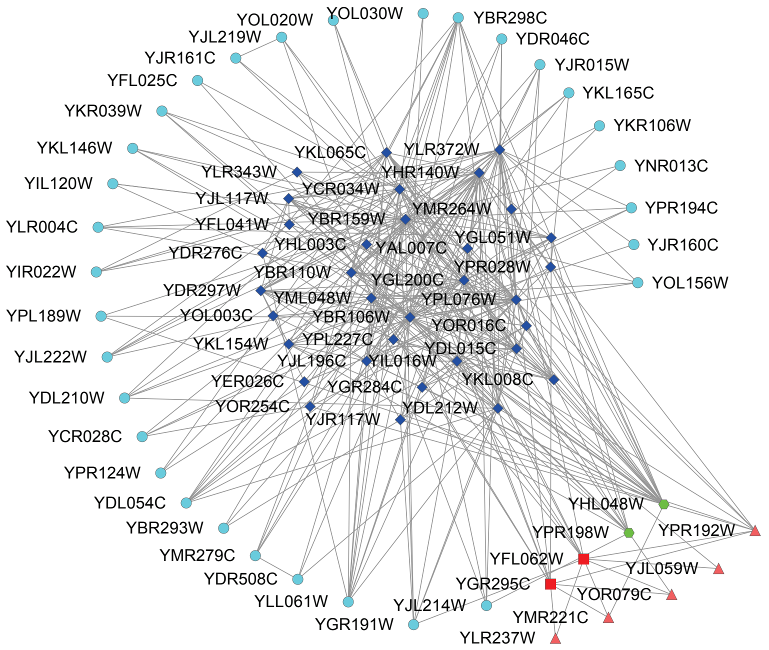

Figure 10: Interactions among functional groups 50, 236, 458 and 631 detected by RSRGM in BioGRID network. Proteins are labeled according to group to which they belong: group 50 (diamond), group 236 (triangle), group 458 (circle), group 631 (hexagon). Proteins YFL062W and YGR295C shared by groups 458 and 631 are labeled with rectangle. Groups 236, 458 and 631 are three identified non-cohesive functional units. This figure is plotted with software Cytoscape [1].

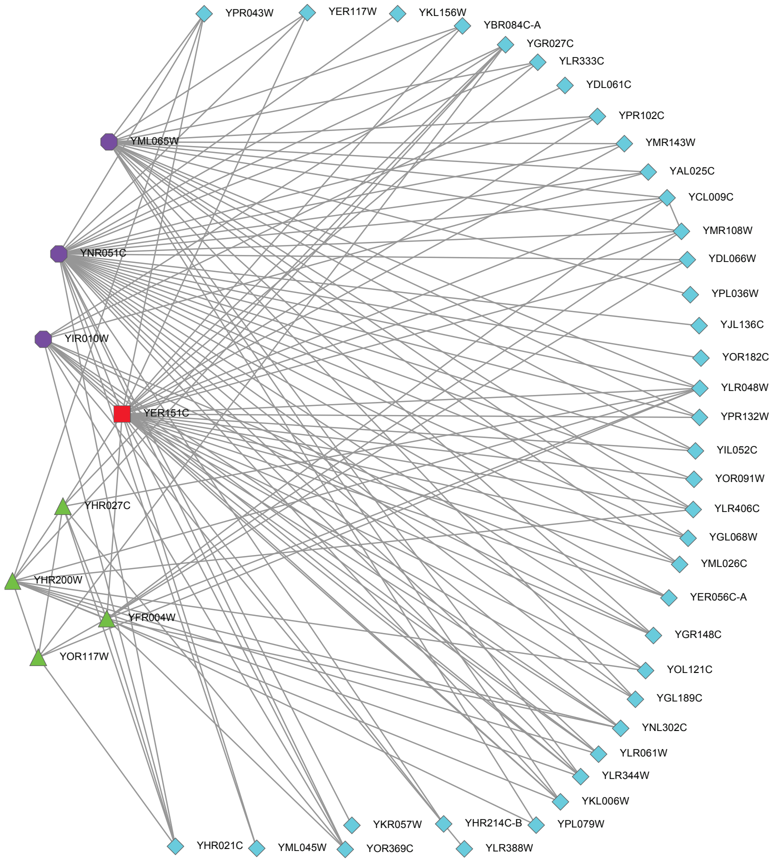

Figure 11: Interactions among functional groups 315, 331 and 555 detected by RSRGM in BioGRID network. Proteins are labeled according to group to which they belong: group 315 (octagon), group 331 (triangle), group 555 (diamond). Proteins YER151C shared by group 315 and 331 is labeled with rectangle. Groups 315 and 555 are two identified non-cohesive functional units. This figure is plotted with software Cytoscape [1].

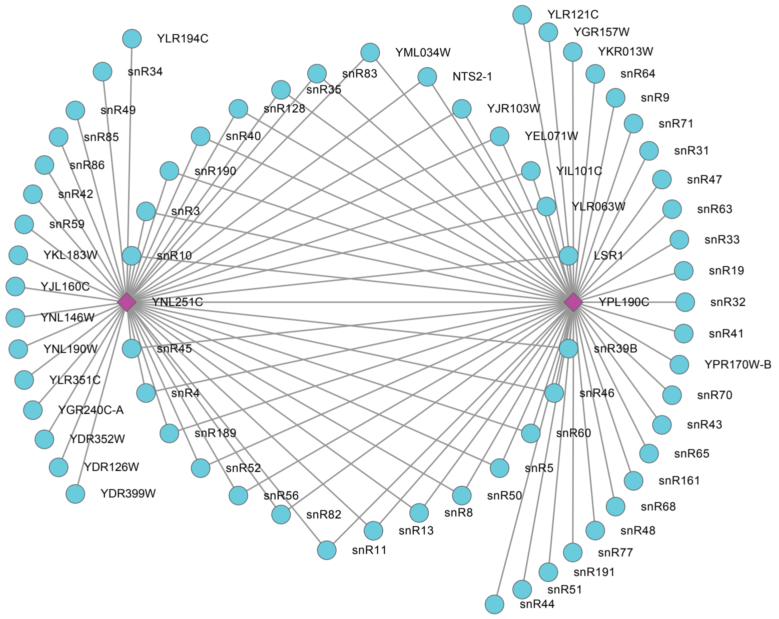

Figure 12: Interactions among functional group 635 detected by RSRGM and two hub proteins YNL251C and YPL190C in BioGRID network. Proteins belonging to group 635 are labeled with circle, and the two hub proteins are labeled with diamond. Group 635 is an identified non-cohesive functional unit. This figure is plotted with software Cytoscape [1].

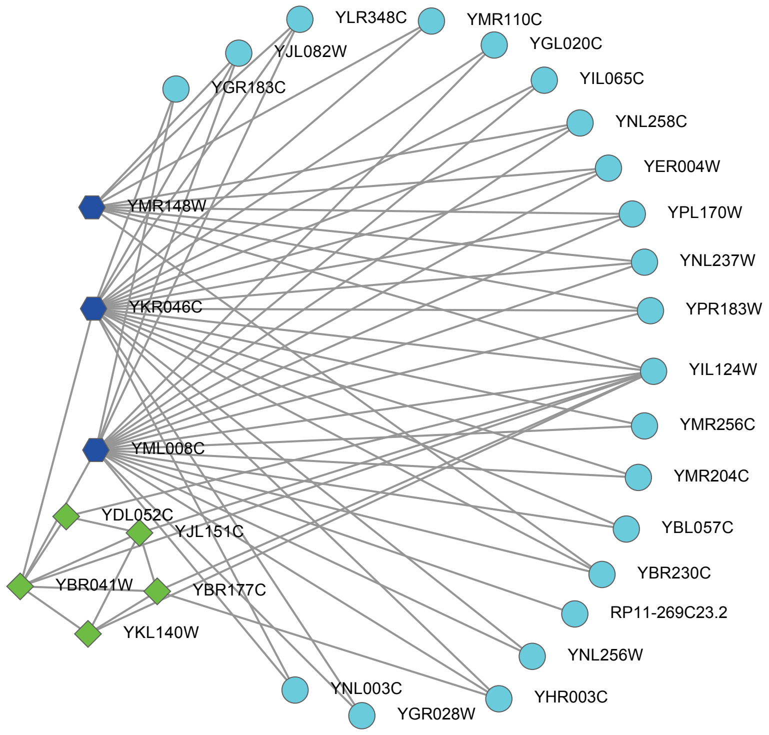

Figure 13: Interactions among functional groups 155, 365 and 685 detected by RSRGM in BioGRID network. Proteins are labeled according to group to which they belong: group 155 (hexagon), group 365(diamond), group 685 (circle). Groups 115 and 685 are two identified non-cohesive functional units. This figure is plotted with software Cytoscape [1].
